# Mitochondrial membrane-based initial separation of MIWI and MILI functions during pachytene piRNA biogenesis

**DOI:** 10.1093/nar/gky1281

**Published:** 2018-12-22

**Authors:** Deqiang Ding, Jiali Liu, Kunzhe Dong, Ashley F Melnick, Keith E Latham, Chen Chen

**Affiliations:** 1 Department of Animal Science, Michigan State University, East Lansing, MI 48824, USA; 2 State Key Laboratory of Agrobiotechnology, College of Biological Sciences, China Agricultural University, Beijing 100193, China; 3 USDA Agricultural Research Service, Avian Disease and Oncology Laboratory, East Lansing, MI 48823, USA; 4 Reproductive and Developmental Sciences Program, Michigan State University, East Lansing, MI 48824, USA; 5 Department of Obstetrics, Gynecology and Reproductive Biology, Michigan State University, Grand Rapids, MI 49503, USA

## Abstract

PIWI-interacting RNAs (piRNAs) engage PIWI proteins to silence transposons and promote germ cell development in animals. In diverse species, piRNA biogenesis occurs near the mitochondrial surface, and involves mitochondrial membrane-anchored factors. In mice, two cytoplasmic PIWI proteins, MIWI and MILI, receive processed pachytene piRNAs at intermitochodrial cement (IMC). However, how MIWI and MILI are initially recruited to the IMC to engage multiple steps of piRNA processing is unclear. Here, we show that mitochondria-anchored TDRKH controls multiple steps of pachytene piRNA biogenesis in mice. TDRKH specifically recruits MIWI, but not MILI, to engage the piRNA pathway. It is required for the production of the entire MIWI-bound piRNA population and enables trimming of MILI-bound piRNAs. The failure to recruit MIWI to the IMC with TDRKH deficiency results in loss of MIWI in the chromatoid body, leading to spermiogenic arrest and piRNA-independent retrotransposon LINE1 de-repression in round spermatids. Our findings identify a mitochondrial surface-based scaffolding mechanism separating the entry and actions of two critical PIWI proteins in the same piRNA pathway to drive piRNA biogenesis and germ cell development.

## INTRODUCTION

P-element induced wimpy testis (PIWI)-interacting RNAs (piRNAs) are a major class of small regulatory RNAs that plays evolutionarily conserved roles in the animal germline to suppress harmful transposons and promote germ cell development (1–7). PIWI proteins are effector proteins guided by bound piRNAs (24–32nt) to impose sequence-specific regulation of gene expression in germ cells (8–12). In diverse species, different PIWI orthologs associate with distinct piRNA populations during different stages of germ cell development to fulfill specific functions. In mice, three PIWI proteins (MIWI, MILI and MIWI2) associate with two distinct developmental stage-specific piRNA populations (13). In fetal/neonatal germ cells, MILI and MIWI2 associate with transposon sequence-rich fetal piRNAs to suppress transposable elements and maintain germline genome integrity (11,12,14). In postnatal germ cells, MIWI and MILI associate instead with transposon sequence-poor pachytene piRNAs that are expressed beginning in the pachytene stage of meiosis to regulate meiotic and postmeiotic gene expression (15–17). Pachytene piRNAs are unique to mammals and are crucial for adult spermatogenesis in mice.

The production of pachytene piRNA involves the processing of long piRNA precursor RNAs into short PIWI-bound piRNAs (13,18). PiRNA biogenesis is believed to occur in a specialized structure called intermitochondrial cement (IMC), an electron-dense non-membranous structure located between mitochondria in pachytene spermatocytes, and containing multiple evolutionarily conserved piRNA biogenesis factors (19–21). Genetic evidence indicates the existence of several intermediate steps for pachytene piRNA biogenesis, which include precursor RNA cleavage into short piRNA intermediates, piRNA intermediates 3′ end trimming by trimmer and 3′ end methylation (22–26). Before piRNA 3′ end maturation, piRNA intermediates are loaded into MIWI and MILI, and PNLDC1 has recently been identified as the trimmer essential for the 3′ end trimming of both MIWI- and MILI-piRNAs (22–24). However, how MIWI and MILI are initially recruited to the IMC to engage early steps of piRNA processing and their links to piRNA maturation steps are less understood.

MIWI and MILI are two similar cytoplasmic PIWI proteins containing the same domain architecture. Both are essential for adult spermatogenesis in mice (27,28). Both are highly expressed in pachytene spermatocytes and associate with virtually the same set of pachytene piRNA species (15,29). After meiosis, MIWI and MILI also both localize and concentrate in the chromatoid body (CB), a large solitary electron dense structure for RNA regulation in round spermatids (20,21). MIWI and MILI both carry out important transposon silencing functions in pachytene spermatocytes. However, their roles in round spermatid transposon silencing diverge after meiosis, with MIWI being required for retrotransposon LINE1 silencing, but MILI becoming dispensable for LINE1 repression (8,9).

Tudor and KH domain-containing (TDRKH or TDRD2) is a conserved mitochondrial Tudor domain protein that plays critical roles in piRNA trimming during piRNA 3′ end maturation in multiple species (30–32). In mice, TDRKH tightly associates with the trimmer PNLDC1 to facilitate piRNA trimming during fetal piRNA biogenesis (22–24). Global knockout of *Tdrkh* in mice leads to a piRNA trimming defect and spermatogenic arrest before the pachytene stage of meiotic prophase I (30). But this germ cell arrest occurs earlier than that in *Pnldc1* knockout mice, indicating that TDRKH has additional functions beyond facilitating piRNA trimming (22). In addition to its association with PNLDC1, TDRKH also directly binds to PIWI proteins in diverse species (33–35). In mice, TDRKH preferentially binds to MIWI but to a less extent to MILI in adult testes (36,37). Such differential binding indicates that TDRKH could differentially regulate MIWI and MILI.

Here, we provide novel genetic evidence that the recruiting mechanisms of MIWI and MILI are distinct at the start of piRNA biogenesis. By conditionally ablating TDRKH in postnatal germ cells in mice and *in vitro* cell-based studies, we reveal that TDRKH is a mitochondrial membrane protein that specifically recruits MIWI, but not MILI, to drive the pachytene piRNA biogenesis. TDRKH controls the production of the entire spectrum of MIWI-piRNAs. TDRKH tethers PNLDC1 to mitochondria and is required for MILI-piRNA trimming. Unexpectedly, the preferential recruitment of MIWI by TDRKH to the IMC is also critical for MIWI, but not MILI, localization in the chromatoid body of round spermatids, which in turn is crucial for transposon silencing in haploid germ cells and for spermiogenesis. These results reveal a mitochondrial surface-based scaffolding mechanism that couples specific PIWI protein recruitment and piRNA trimming essential for separable downstream effector functions of different PIWI proteins. These findings also suggest species diversity in mitochondrial TDRKH-mediated assembly of piRNA processing machinery.

## MATERIALS AND METHODS

### Ethics statement

All the animal procedures were approved by the Institutional Animal Care and Use Committee of Michigan State University. All experiments with mice were conducted ethically according to the Guide for the Care and Use of Laboratory Animals and institutional guidelines.

### Mouse strains

*Tdrkh^tm1a^* mice were acquired from the Jackson Laboratory. To generate *Tdrkh^fl^* allele with exon 3 flanked by loxP sites, heterozygous *Tdrkh^tm1a^* animals were bred with FLP-expressing transgenic mice (Jackson Laboratory) to remove FRT flanked sequences. *Tdrkh^fl/+^* males were bred with *Tdrkh^fl/+^* females to generate homozygous *Tdrkh^fl/fl^* mice. To generate Stra8-Cre *Tdrkh* conditional knockout mice (*Tdrkh^cKO^*), *Stra8*-Cre transgenic mice (Jackson Laboratory) were bred with *Tdrkh^fl/fl^* mice. Primers for *Tdrkh^fl/fl^* mice genotyping PCR are: 5′-TGTCAGATGAGAGGCAGCAG-3′ and 5′-CCATTTGGCATTTTCTTTGG-3′. Wild type allele produced a 228 bp product; *Tdrkh^fl^* allele generated a 397bp product. Primers for Stra8-Cre PCR were previously described (29).

*Miwi* knockout mice (*Miwi^−/−^*), generated in the laboratory of Dr. Haifan Lin (27), were purchased from Mutant Mouse Resource Research Centers. *Mov10l1* flox (*Mov10l1^fl/fl^*) mice, generated in the laboratory of Dr. Eric Olson, were purchased from Jackson Laboratory (38). To generate *Mov10l1* conditional knockout mice (*Mov10l1^cKO^*), *Mov10l1^fl/fl^* mice were bred with Stra8-Cre mice (Jackson Laboratory). *Tdrkh* knockout mice (*Tdrkh^tm1b(KOMP)Wtsi^*, also as *Tdrkh^−/−^*) were purchased from Jackson Laboratory. *Pnldc1* knockout mice (*Pnldc1^−/−^*) were generated as described (22).

### Plasmid construction

The full-length mouse *Miwi, Mili, Miwi2* and *Pnldc1* cDNAs were amplified by PCR and cloned into the pEGFP-C1 vector that contains a N-terminal GFP tag. The full-length mouse *Tdrkh* (1–560aa), *Tdrkh-ΔTM* mutant (35–560aa) and *Tdrkh-ΔTud* mutant (D390A/F391A) cDNAs were amplified by PCR and cloned into the pEGFP-N1 vector that contains a C-terminal GFP tag. To obtain the C-terminal RFP-tagged TDRKH, TDRKH-ΔTM and TDRKH-ΔTud plasmids, the indicated cDNAs were cloned into a modified pEGFP-C1 vector with GFP replaced by Red Fluorescent Protein (TurboRFP). To obtain FLAG-tagged TDRKH and PNLDC1 plasimids, the full-length *Tdrkh* and *Pnldc1* cDNAs were cloned into a modified pcDNA3 vector encoding a FLAG-tag.

### Histology

Mouse testes and epididymides were fixed in Bouin's fixative in PBS at 4°C overnight and embedded in paraffin. 5 μm sections were cut and stained with hematoxylin and eosin after dewaxing and rehydration.

### Immunofluorescence

Testes were fixed in 4% PFA in PBS overnight at 4°C and embedded in paraffin. 5 μm sections were cut, dewaxed and rehydrated. Antigen retrieval was performed by microwaving the sections in 0.01 M sodium citrate buffer (pH 6.0). After rinsing with PBS, tissue sections were blocked in 5% normal goat serum (NGS) for 30 min at room temperature (RT). Testis sections were then incubated with anti-MIWI (1:100; 2079, Cell Signaling Technology), anti-MILI (1:100; PM044, MBL), anti-TDRKH (1:100; 13528-1-AP, Proteintech), anti-AIF (1:100; 5318, Cell Signaling Technology), anti-ACRV1 (1:50; 14040-1-AP, Proteintech), anti-GASZ (1:50; 21550-1-AP, Proteintech), anti-MVH (1:100; Ab27591, Abcam), anti-TOMM20 (1:50; sc-17764, Santa Cruz Biotechnology), anti-LINE1 ORF1 (1:800), or FITC-conjugated mouse anti-γH2AX (1:500; 16–202A, Millipore) in 5% NGS at 37°C for 2 h. After washing with PBS, sections were incubated with Alexa Fluor 555 goat anti-rabbit IgG (1:500; A21429, Life Technologies) and/or Alexa Fluor 488 goat anti-mouse IgG (1:500; A11029, Life Technologies) for 1 h at RT and mounted using Vectorshield mounting media with DAPI (H1200, Vector Laboratories) after washing. Fluorescence microscopy was performed using Fluoview FV1000 confocal microscope (Olympus, Japan).

### HeLa cell immunofluorescence

HeLa cells were transfected with indicated plasmids. Forty eight hours after transfection, the cells were stained with 100 nM Mitotracker Red Probes (M7512, Thermo Scientific) at 37°C for 30 min and fixed with 3.7% formaldehyde at 37°C for 15 min. After washing with PBS, cells were mounted using Vectorshield mounting media with DAPI (H1200, Vector Laboratories). Fluorescence microscopy was performed using Fluoview FV1000 confocal microscope (Olympus, Japan).

### Transmission electron microscopy

Mouse testes were fixed in 0.1 M cacodylate buffer supplemented with 2.5% glutaraldehyde and 2% PFA for 2 h at room temperature. After washing with 0.1 M cacodylate buffer, the testes were post-fixed in 0.1 M cacodylate buffer supplemented with 1% osmium tetroxide for 2 h at room temperature. After washing, the testes were dehydrated in increasing concentrations of acetone and then infiltrated and embedded in Spurr. 70 nm thin sections were obtained with a Power Tome Ultramicrotome (Boeckeler Instruments, USA) and post stained with uranyl acetate and lead citrate. Images were taken with JEOL 100CX Transmission Electron Microscope (Japan Electron Optics Laboratory, Japan) at an accelerating voltage of 100kV.

### Western blotting

Mouse testes were collected and homogenized using RIPA buffer (50 mM Tris·HCl pH 7.4, 1% NP-40, 0.5% sodium deoxycholate, 0.01% SDS, 1 mM EDTA, and 150 mM NaCl). Protein lysates were separated by 4–20% SDS-PAGE gel and transferred to PVDF membranes (Bio-Rad). The membranes were blocked in 5% non-fat milk and subsequently incubated with primary antibodies in blocking solution overnight at 4°C. Membranes were washed with TBST and incubated with HRP-conjugated goat anti-rabbit IgG (1:5000; 1706515, Bio-Rad) or goat anti-mouse IgG (1:5000; 1706516, Bio-Rad) for 1 h before chemiluminescent detection. The primary antibodies used were: anti-TDRKH (1:4000; 13528-1-AP, Proteintech), anti-MIWI (1:1000; 2079, Cell Signaling Technology), anti-MILI (1:2000; PM044, MBL), and HRP-conjugated mouse anti-β-actin (1:5000; A3854, Sigma).

### Co-Immunoprecipitation

HEK293T cells were transfected with indicated plasmids using Lipofectamine 2000 (Life Technologies). After 48 h, the cell lysates were treated with or without RNase A (500 μg/ml) for 3 h at 4°C followed by 30 min at room temperature. Immunoprecipitation were performed using anti-FLAG M2 Affinity Gel (A2220, Sigma). FLAG-tagged or GFP-tagged proteins were detected by Western blotting using anti-FLAG antibody (1:1000; F1804, Sigma) and anti-GFP antibody (1:10 000; Ab290, Abcam).

### Immunoprecipitation of piRNAs

Mouse testes were collected and homogenized using lysis buffer (20 mM HEPES pH 7.3, 150 mM NaCl, 2.5 mM MgCl_2_, 0.2% NP-40 and 1 mM DTT) with protease inhibitor cocktail (Thermo Scientific) and RNase inhibitor (Promega). After sonication, testis lysates were centrifuged at 12 000 rpm for 10 min. The supernatants were pre-cleared using protein-A agarose beads (Roche) for 2 h. Anti-MILI (PM044, MBL) or anti-MIWI (2079, Cell Signaling Technology) antibodies together with protein-A agarose beads were added to the lysates and incubated for 4 h. The beads were washed in lysis buffer for five times. Immunoprecipitated RNAs were isolated from the beads using Trizol reagent (Thermo Scientific) for piRNA labeling or small RNA library construction. For protein detection, immunoprecipitated beads were boiled in protein loading buffer for 5 min. Western blotting of MILI or MIWI was performed as described above. For small RNA library construction, total MILI or MIWI protein amount was matched between wild-type and *Tdrkh^cKO^* samples after immunoprecipitation for RNA isolation to ensure that sequencing results are directly comparable.

### Detection of piRNAs

Total RNA was extracted from mouse testes using Trizol reagent (Thermo Scientific). Total RNA (1 μg) or immunoprecipitated RNA (MILI or MIWI) was de-phosphorylated with Shrimp Alkaline Phosphatase (NEB) and end-labeled using T4 polynucleotide kinase (NEB) and [γ-^32^P] ATP. The ^32^P-labeled RNA was separated on a 15% urea–PAGE gel, and signals were detected by exposing the gel on phosphorimager screen followed by scanning on the Typhoon scanner (GE Healthcare).

### Small RNA libraries and bioinformatics

Small RNA libraries from immunoprecipitated RNAs or total RNA were prepared using NEBNext® Multiplex Small RNA Library Prep Kit (E7300, NEB) following manufacturer's instructions. Multiple libraries with different barcodes were pooled and sequenced with the Illumina HiSeq 4000 platform (MSU Genomic Core Facility).

Sequenced reads were processed with fastx_clipper (http://hannonlab.cshl.edu/fastx_toolkit/index.html) to clip the sequencing adapter read-through. Clipped reads were filtered by length (24–48 nt) and aligned to the following sets of sequences: piRNA clusters, coding RNAs, non-coding RNAs, repeats and intron. Reads not mapping to the above five sets of sequences were classified as ‘other’. Alignments were performed with Bowtie (one base mismatch allowed). Repeats included classes of repeats as defined by RepeatMasker (ftp://hgdownload.cse.ucsc.edu/goldenPath/mm10/database/rmsk.txt.gz).

### 5′-5′ and 3′-3′ distance analysis of piRNAs and piRNA intermediates

24–48 nt reads from wild-type or *Tdrkh^cKO^* MILI-piRNA libraries were separately mapped to piRNA clusters. Only perfect match was considered. The top 10000 distinctively mapped piRNAs from wild-type MILI-piRNA libraries were extracted and were used as position references. For 5′–5′ distance analysis of piRNA-piRNA intermediate, the 5′ end positions of top 10 000 piRNA references were defined as position 0; the density of mapped piRNA 5′ ends from *Tdrkh^cKO^* MILI-piRNA libraries in a window of 60 nt (-30 nt→+30 nt) was measured. Frequency of piRNA 5′ ends at each position was plotted. For 3′–3′ distance analysis of piRNA-piRNA intermediate, the 3′ end positions of top 10 000 piRNA references were defined as position 0; the density of mapped piRNA 3′ ends from *Tdrkh^cKO^* MILI-piRNA libraries in a window of 60 nt (-30 nt→+30 nt) was measured. Frequency of piRNA 3′ ends at each position was plotted.

## RESULTS

### Conditional deletion of *Tdrkh* in postnatal germ cells leads to spermiogenic arrest

**Figure 1.**
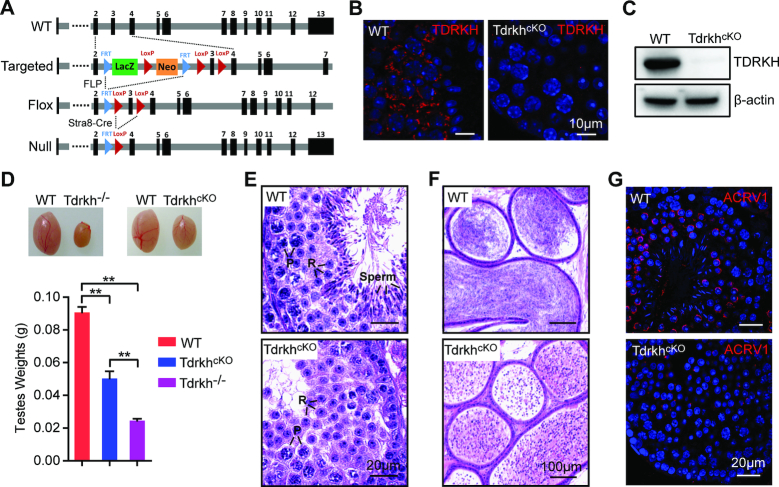
Loss of TDRKH in postnatal male germ cells causes spermatogenic arrest in mice. (**A**) A schematic diagram showing the gene targeting strategy for the generation of a *Tdrkh* conditional allele. Cre-mediated deletion removed the exon3 of *Tdrkh* and generated a protein null allele. (**B**) Immunostaining of TDRKH in adult WT and *Tdrkh^cKO^* testes. DNA was stained with DAPI. Scale bar, 10 μm. (**C**) Western blotting of TDRKH expression in adult WT and *Tdrkh^cKO^* testes. β-Actin served as a loading control. (**D**) Testicular atrophy in *Tdrkh^cKO^* mice. Testis sizes and weights of adult WT, *Tdrkh^cKO^* and *Tdrkh*^*−/*^*^−^* mice are shown. *n* = 8. *Error bars* represent s.e.m. The *P*-value was calculated using unpaired *t*-test. **P < 0.01. (**E**) Spermatogenic arrest in *Tdrkh^cKO^* testes. Hematoxylin and eosin stained testis sections from adult WT and *Tdrkh^cKO^* mice are shown. Scale bars, 20 μm. P, pachytene spermatocytes; R, round spermatids; Sperm, spermatozoa. (**F**) Hematoxylin and eosin stained epididymis sections from adult WT and *Tdrkh^cKO^* mice are shown. Scale bars, 100 μm. (**G**) Spermatogenic arrest at the round spermatid stage in *Tdrkh^cKO^* testes. Co-immunostaining of ACRV1 and γH2AX in stage VII–VIII seminiferous tubule from WT and *Tdrkh^cKO^* testes. DNA was stained with DAPI. Scale bar, 20 μm.

To define the specific involvement of TDRKH in PIWI regulation and pachytene piRNA biogenesis in postnatal germ cells, we generated *Tdrkh* conditional knockout (*Tdrkh^cKO^*) mice in which *Tdrkh* was deleted in prospermatogonia starting at postnatal Day 3 by *Stra8*-Cre transgene (29,39). We used a *Tdrkh* conditional allele (*Tdrkh^fl^*) with the exon 3 of *Tdrkh* flanked by two loxP sites. This allele was derived from a targeted ‘knockout-first’ allele by FLP mediated recombination (Figure 1A). By combining *Tdrkh^fl^* with *Stra8*-Cre, we obtained *Stra8*-Cre^+^, *Tdrkh^fl/-^* conditional knockout mice with *Tdrkh* deletion in all adult germ cell lineages. We confirmed the successful inactivation of TDRKH by immunofluorescence and Western blotting, which showed absence of TDRKH protein in adult germ cells and the testes, respectively (Figure 1B and C). *Tdrkh^cKO^* mice exhibited smaller testes with an average of 50% of wild-type control testis weight (Figure 1D). The *Tdrkh^cKO^* testis weight is two-fold higher than that of *Tdrkh^−/^*^*−*^ mice, suggesting that *Tdrkh^cKO^* germ cells arrest at a later stage than *Tdrkh^−/−^* mice. Histological examination of *Tdrkh^cKO^* testis sections revealed that germ cells were primarily arrested at the round spermatid stage (80%), with 20% seminiferous tubules arrested at the zygotene spermatocyte stage (Figure 1E and Supplementary Figure S1). This contrasts with *Tdrkh^−/^*^*−*^ mice, which display complete meiotic arrest at the zygotene stage (22,30). Analysis of *Tdrkh^cKO^* epididymis showed numerous round spermatid-like cells (Figure 1F). Further examination indicates that *Tdrkh^cKO^* round spermatids arrested before step 5 because only proacrosome granules but not acrosome caps were observed in arrested spermatids (Figure 1G). Together, these results indicate that postnatal expression of TDRKH is critical for spermatid development and spermiogenesis.

### TDRKH is required for pachytene piRNA biogenesis

**Figure 2.**
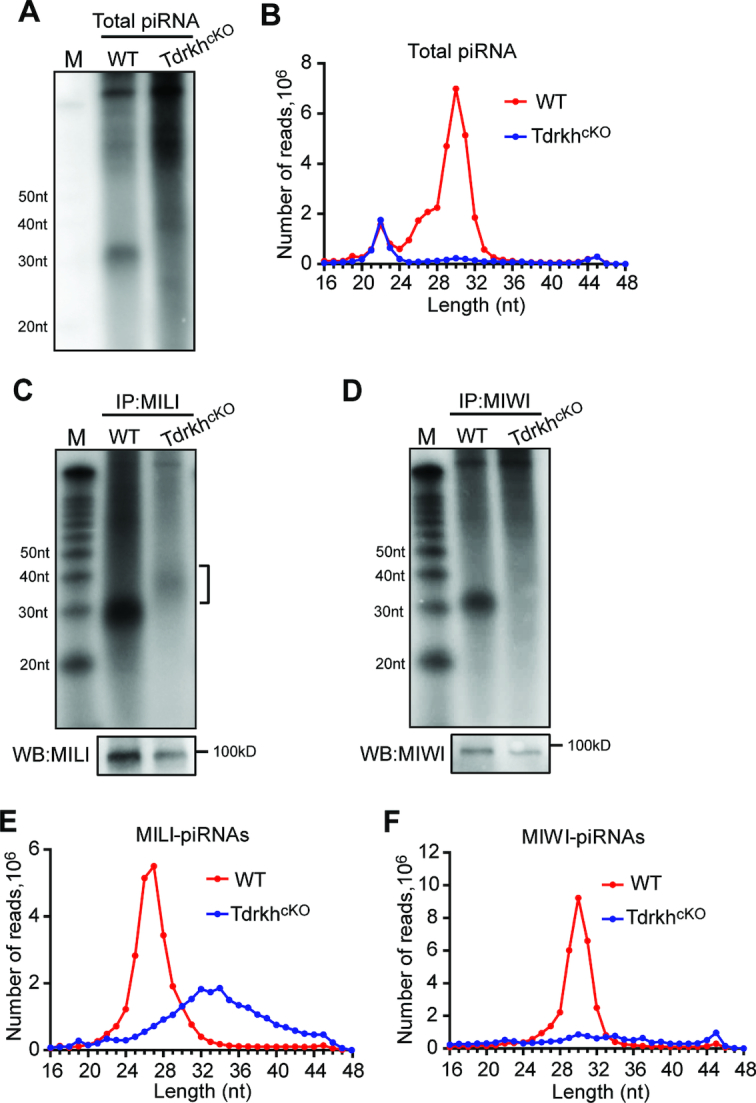
Increased piRNA lengths and reduced piRNA levels in adult *Tdrkh^cKO^* testes. (**A**) Severe reduction of total piRNAs in *Tdrkh^cKO^* testes. Total RNA from adult WT and *Tdrkh^cKO^* testes were end-labeled with [^32^P]-ATP and detected by 15% TBE urea gel and autoradiography. (**B**) The length distribution of small RNAs from adult WT and *Tdrkh^cKO^* testicular small RNA libraries. Data were normalized by miRNA reads (21–23nt). (**C**) Extension of MILI-piRNAs in *Tdrkh^cKO^* testes. Small RNAs were isolated from immunoprecipitated MILI-RNPs, end-labeled with [^32^P]-ATP and detected by 15% TBE urea gel and autoradiography. Western blotting was performed using anti-MILI antibody to show immunoprecipitation efficiency. Square bracket indicates extended piRNAs. M: molecular weight marker. (**D**) Diminished MIWI-piRNAs in *Tdrkh^cKO^* testes. Small RNAs were isolated from immunoprecipitated MIWI-RNPs, end-labeled with [^32^P]-ATP and detected by 15% TBE urea gel and autoradiography. Western blotting was performed using anti-MIWI antibody to show immunoprecipitation efficiency. M: molecular weight marker. (**E**) The length distribution of MILI-piRNAs from adult WT and *Tdrkh^cKO^* MILI-piRNA libraries. (**F**) The length distribution of MIWI-piRNAs from adult WT and *Tdrkh^cKO^* MIWI-piRNA libraries.

To define the role of TDRKH in pachytene piRNA biogenesis, we examined the abundance and size of piRNA populations in adult wild-type and *Tdrkh^cKO^* testes. Radiolabeling of total small RNAs revealed an expected wild-type piRNA population around 30nt in length, however, this piRNA population was absent in *Tdrkh^cKO^* testes (Figure 2A). Sequencing of small RNA libraries constructed from total RNA confirmed the near depletion of the whole piRNA population in *Tdrkh^cKO^* testes after normalized with miRNA counts (Figure 2B).

To assess whether there are still residual piRNAs associated with PIWI proteins in *Tdrkh^cKO^* testes, we immunoprecipitated MILI and MIWI and labeled associated small RNAs. In *Tdrkh^cKO^* testes, MILI associated with a population of small RNAs with extended lengths and with much lower abundance as compared to wild-type (Figure 2C). Strikingly, MIWI did not associate with any detectable piRNAs in *Tdrkh^cKO^* testes (Figure 2D). Small RNA sequencing of libraries constructed from immunoprecipitated small RNAs confirmed these observations that MILI still associates with small amount of extended small RNAs while MIWI bound piRNAs are completely absent (Figure 2E and F). These data indicate that postnatal TDRKH is essential for pachytene piRNA production with a differential effect on MILI-bound and MIWI-bound piRNAs.

### TDRKH is required for 3′ trimming of MILI-piRNAs

**Figure 3.**
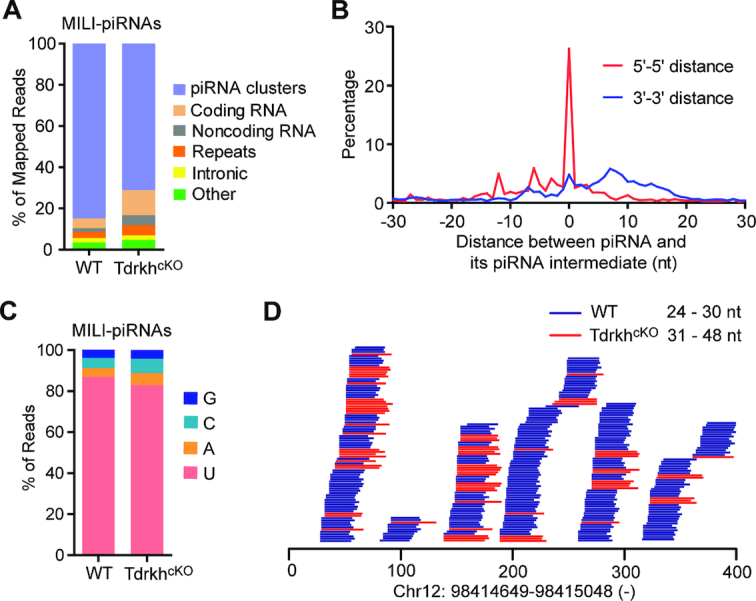
The 3′ end extension of MILI-piRNAs in adult *Tdrkh^cKO^* testes. (**A**) Genomic annotation of MILI-piRNAs from adult WT and *Tdrkh^cKO^* testes. Sequence reads (24–48 nt) from MILI-piRNAs libraries were aligned to mouse genomic sequence sets in the following order: piRNA clusters, coding RNA, non-coding RNA, repeats, intronic sequences and other. The percentage of mapped reads is shown. (**B**) Density plot of 5′–5′ and 3′–3′ distances between WT piRNAs and *Tdrkh^cKO^* piRNA intermediates. The 24–48nt reads from WT and *Tdrkh^cKO^* MILI-piRNA libraries were mapped to piRNA clusters. The Top 10000 distinctively mapped piRNAs from WT MILI-piRNA library were extracted as references. The frequency distribution of distances of 5′ ends of mapped reads from *Tdrkh^cKO^* MILI-piRNA library and referenced piRNA 5′ ends (5′–5′ distance) and the frequency distributions of distances of 3′ ends of mapped reads from *Tdrkh^cKO^* MILI-piRNA library and referenced piRNA 3′ end (3′–3′ distance) are shown. (**C**) Nucleotide distributions at the first position in MILI-piRNAs from adult WT and *Tdrkh^cKO^* testes. 24–48nt reads from MILI-piRNAs libraries were used. (**D**) Extended piRNA 3′ ends in *Tdrkh^cKO^* testes. Alignments between 31–48 nt reads from *Tdrkh^cKO^* MILI-piRNA library and 24–30 nt reads from WT MILI-piRNA library within a selected region from a representative piRNA cluster. The genomic location of the piRNA cluster is shown at the bottom.

To understand the characteristics of the residual extended *Tdrkh^cKO^* small RNA population bound to MILI, we constructed and sequenced small RNA libraries generated from RNA extracted from immunoprecipitated MILI in wild-type and *Tdrkh^cKO^* testes. We mapped 24–48nt reads from both wild-type and *Tdrkh^cKO^* MILI-piRNA libraries to the mouse genome (22) and observed that, like wild-type, *Tdrkh^cKO^* MILI-piRNAs were primarily mapped to piRNA clusters (Figure 3A). This indicates that *Tdrkh^cKO^* MILI-RNAs are extended piRNAs, consistent with a role of TDRKH in pachytene piRNA trimming. To confirm TDRKH is responsible for piRNA 3′ end trimming, we further compared 5′–5′ and 3′–3′ distances between wild-type and *Tdrkh^cKO^* MILI-piRNAs. The frequency distribution plots showed a predominant single peak at position 0 for 5′–5′ distance, indicating that *Tdrkh^cKO^* MILI-RNAs share the same 5′ ends with the wild-type MILI-piRNAs (Figure 3B). The 3′-3′ distance plot showed that the 3′ ends of *Tdrkh^cKO^* MILI-piRNAs extended to downstream but not upstream of wild-type MILI-piRNAs, indicating *Tdrkh^cKO^* MILI-piRNAs are untrimmed at 3′ ends (Figure 3B). Consistent with this result, the first nucleotides of *Tdrkh^cKO^* MILI-piRNAs exhibited the same strong U bias (Figure 3C). When mapping to specific piRNA clusters, *Tdrkh^cKO^* MILI-piRNAs matched the 5′ ends of wild-type piRNAs, but displayed 3′ end extensions, indicating defective piRNA 3′ trimming in *Tdrkh^cKO^* mice (Figure 3D). Such piRNA defect was also observed in *Pnldc1^−/−^* mice (Supplementary Figure S2). These results demonstrate that, similar to its important role in pre-pachytene piRNA trimming, TDRKH also participates in pachytene piRNA 3′ end trimming during adult spermatogenesis.

### Complete loss of MIWI-piRNAs in *Tdrkh^cKO^* testes

*Tdrkh^cKO^* MILI-piRNAs are significantly reduced in abundance and are untrimmed, it remains unclear why MIWI-piRNAs in *Tdrkh^cKO^* were undetectable. To study the specific mechanism by which TDRKH participates in MIWI-piRNA production, we compared MIWI expression and the piRNA defect in *Tdrkh^cKO^* mice with those in *Pnldc1^−/−^* mice (22) and *Stra8*-Cre^+^, *Mov10l1^fl/-^* conditional knockout (*Mov10l1^cKO^*) mice (29). These two piRNA biogenesis factors are critical for MIWI-piRNA production. MIWI protein expression in *Tdrkh^cKO^* testes was reduced compared to wild-type and the level of reduction was similar to that in *Pnldc1^−/−^* and *Mov10l1^cKO^* testes (Supplementary Figure S3). PNLDC1 is required for pachytene piRNA 3′ end trimming (22–24). *Pnldc1^−/−^* testes showed piRNA length extension and reduced piRNA levels. However, the piRNA reduction in *Pnldc1^−/−^* testes is milder than that in *Tdrkh^cKO^* testes, as extended MILI-piRNAs and MIWI-piRNAs were both observed in *Pnldc1^−/−^* testes (22). MOV10L1 is a RNA helicase required for piRNA processing (40–43). *Mov10l1^cKO^* testes showed reduced piRNA level similar to that of *Tdrkh^cKO^* testes (Supplementary Figure S4A and D). However, we still observed residual MILI-piRNAs (Supplementary Figure S4B and E) and MIWI-piRNAs (Supplementary Figure S4C and F) in *Mov10l1^cKO^* testes. As a result, reduced MIWI protein level and defective piRNA 3′ end trimming in *Tdrkh^cKO^* testes cannot fully explain why MIWI-piRNAs in *Tdrkh^cKO^* are completely absent.

### TDRKH is required for MIWI but not MILI localization to the IMC

**Figure 4.**
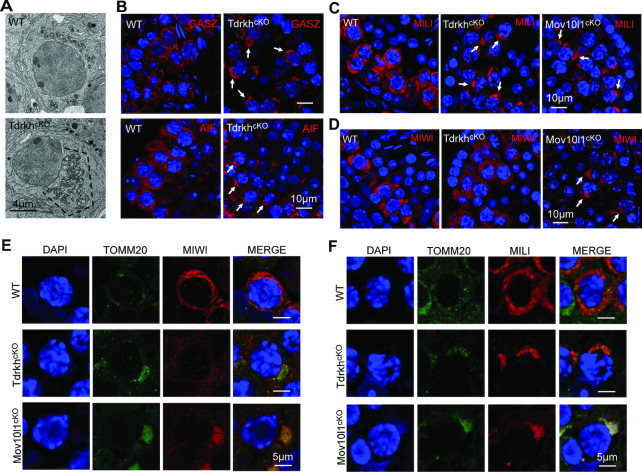
TDRKH deficiency selectively disrupts the localization of MIWI to mitochondria in spermatocytes. (**A**) Loss of TDRKH causes the aggregation of mitochondria in spermatocytes. Transmission electron microscopy was performed on pachytene spermatocytes from adult WT and *Tdrkh^cKO^* testes. The mitochondria aggregation is indicated by dotted line. Scale bar, 4 μm. (**B**) TDRKH deficiency causes the aggregation of mitochondrial proteins GASZ and AIF in spermatocytes. Immunostaining was performed using indicated antibodies on adult WT and *Tdrkh^cKO^* testes. DNA was stained with DAPI. Protein aggregations are indicated by arrows. Scale bar, 10 μm. (**C, D**) TDRKH deficiency causes the aggregation of MILI (C), but not MIWI (D), in spermatocytes. Immunostaining was performed using indicated antibodies on adult WT, *Tdrkh^cKO^* and *Mov10l1^cKO^* testes. DNA was stained with DAPI. Protein aggregations are indicated by arrows. Scale bar, 10 μm. (**E**) Loss of TDRKH disrupts the localization of MIWI to mitochondria. Co-immunostaining was performed using MIWI and TOMM20 antibodies on adult WT, *Tdrkh^cKO^* and *Mov10l1^cKO^* testes. DNA was stained with DAPI. TOMM20 served as a mitochondrial marker. Scale bar, 5 μm. (**F**) The congregation of MILI in *Tdrkh^cKO^*and *Mov10l1^cKO^*testes. Co-immunostaining was performed using MILI and TOMM20 antibodies on adult WT, *Tdrkh^cKO^* and *Mov10l1^cKO^* testes. DNA was stained with DAPI. TOMM20 served as a mitochondrial marker. Scale bar, 5 μm.

Next, we examined the localization of MIWI and MILI in *Tdrkh^cKO^* testes. TDRKH is a mitochondrial localized protein in male germ cells (Supplementary Figure S5) (30). We observed that *Tdrkh^cKO^* spermatocytes displayed a polar distribution of extensively clustered mitochondria aggregates (polar conglomeration) by electron microscopy (Figure 4A), a phenotype also observed in *Pnldc1^−/−^* and *Mov10l1^cKO^* mice (22,24,42). Immunofluorescence of GASZ and AIF, a mitochondrial localized piRNA factor and a mitochondrial marker, respectively, confirmed the polar clustering of mitochondria in *Tdrkh^cKO^* spermatocytes (Figure 4B). Consistent with mitochondrial clustering, MILI was localized at aggregated mitochondria in *Tdrkh^cKO^* spermatocytes, resembling GASZ and AIF localization pattern (Figure 4C). Interestingly, MIWI localization was different from MILI in *Tdrkh^cKO^* spermatocytes, with diffused expression throughout cytoplasm (Figure 4D). In sharp contrast, MIWI localization in *Mov10l1^cKO^* spermatocytes was restricted to clustered mitochondria (Figure 4D). This suggests that TDRKH deficiency selectively affects the localization MIWI but not MILI to IMC. To further test effects of TDRKH deficiency on MIWI and MILI localization, we co-immunostained MIWI and TOMM20 (a mitochondrial marker) or MILI and TOMM20 on wild-type, *Tdrkh^cKO^*, and *Mov10l1^cKO^* spermatocytes. MIWI was selectively dispersed in *Tdrkh^cKO^* but not in *Mov10l1^cKO^* spermatocytes despite apparent mitochondrial clustering in both mutant cells (Figure 4E and Supplementary Figure S6). This is in contrast with MILI colocalization with TOMM20 in both *Tdrkh^cKO^* and *Mov10l1^cKO^* spermatocytes (Figure 4F and Supplementary Figure S7). These results indicate that TDRKH is required for MIWI but not MILI localization to IMC. This is consistent with the *in vitro* finding that MILI interacts with TDRKH to a less extent than MIWI (30,36) (Supplementary Figure S8), suggesting the regulation of MIWI and MILI are biochemically and genetically separable.

### Membrane-anchored TDRKH recruits MIWI to mitochondria

**Figure 5.**
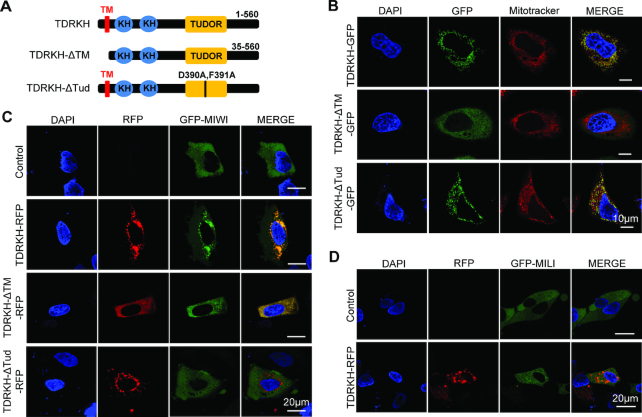
Mitochondria-anchored TDRKH recruits MIWI but not MILI to mitochondria. (**A**) Domain architecture of TDRKH and its mutations. TM, transmembrane domain. Tud, Tudor domain. (**B**) TDRKH anchors to mitochondria through its transmembrane region. HeLa cells were transfected with indicated GFP-tagged plasmids. After 48 h, the transfected cells were stained with Mitotracker and then fixed. DNA was stained with DAPI. Scale bar, 10 μm. (**C**) TDRKH recruits MIWI to mitochondria. HeLa cells were transfected with GFP tagged MIWI plasmids, along with indicated RFP tagged plasmids. After 48 h, the cells were fixed and DNA was stained with DAPI. Scale bar, 20 μm. (**D**) TDRKH does not recruit MILI to mitochondria. HeLa cells were transfected with GFP tagged MILI plasmids, along with indicated RFP tagged plasmids. After 48 h, the cells were fixed and DNA was stained with DAPI. Scale bar, 20 μm.

We next tested whether TDRKH could directly recruit MIWI to mitochondria. TDRKH is a mitochondrial localized protein with a putative mitochondrial localization sequence (MLS) at the N-terminus and two RNA binding KH domains and a protein binding Tudor domain (30,36) (Figure 5A and Supplementary Figure S5). The dependence of TDRKH on MLS for mitochondrial localization has not been formally proven. We used a HeLa cell-based heterologous expression system in which we expressed TDRKH-GFP and a mutant TDRKH with a transmembrane domain deletion (TDRKH-ΔTM-GFP). TDRKH-GFP colocalized with mitotracker on mitochondria whereas TDRKH-ΔTM-GFP were diffused in cytoplasm, indicating that the MLS is responsible for autonomous TDRKH localization on mitochondrial membrane (Figure 5B). Therefore, TDRKH is a mitochondrial protein that autonomously anchors on mitochondrial membrane through its transmembrane domain. We then examined whether mitochondrial anchored TDRKH could recruit MIWI or MILI to mitochondria. We constructed and expressed GFP-MIWI or GFP-MILI, both showed diffused distribution in cytoplasm when singly transfected into HeLa cells (Figure 5C and D). When co-expressed with TDRKH-RFP, GFP-MIWI concentrated on mitochondria to significantly overlap with TDRKH-RFP, suggesting the direct recruitment of MIWI by mitochondrial anchored TDRKH (Figure 5C). The deletion of TDRKH MLS signal dispersed TDRKH mitochondrial localization and resulted in corresponding GFP-MIWI diffusion (Figure 5C). In contrast, co-expression of TDRKH-RFP and GFP-MILI did not result in MILI mitochondrial accumulation, suggesting the inability of mitochondria-anchored TDRKH to recruit MILI (Figure 5D). To determine whether MIWI recruitment by TDRKH to mitochondria is mediated by direct protein-proteins interactions, we co-expressed a TDRKH Tudor domain mutant (D390A/F391A, we refer to as TDRKH-ΔTud) with MIWI. This Tudor domain mutation disrupts the TDRKH interaction with MIWI (36). TDRKH-ΔTud-GFP colocalized with mitotracker on mitochondria in HeLa cells, indicating that Tudor domain mutation does not affect the TDRKH localization (Figure 5B). However, co-expression of TDRKH-ΔTud-RFP and GFP-MIWI did not allow MIWI mitochondrial accumulation (Figure 5C). Together, these data indicate that TDRKH selectively recruits MIWI but not MILI to mitochondria through direct TDRKH-MIWI protein interaction. This is consistent with the above observation that MIWI but not MILI localization to IMC was disrupted by TDRKH loss in *Tdrkh^cKO^* spermatocytes.

### TDRKH is required for MIWI but not MILI localization to the chromatoid body

**Figure 6.**
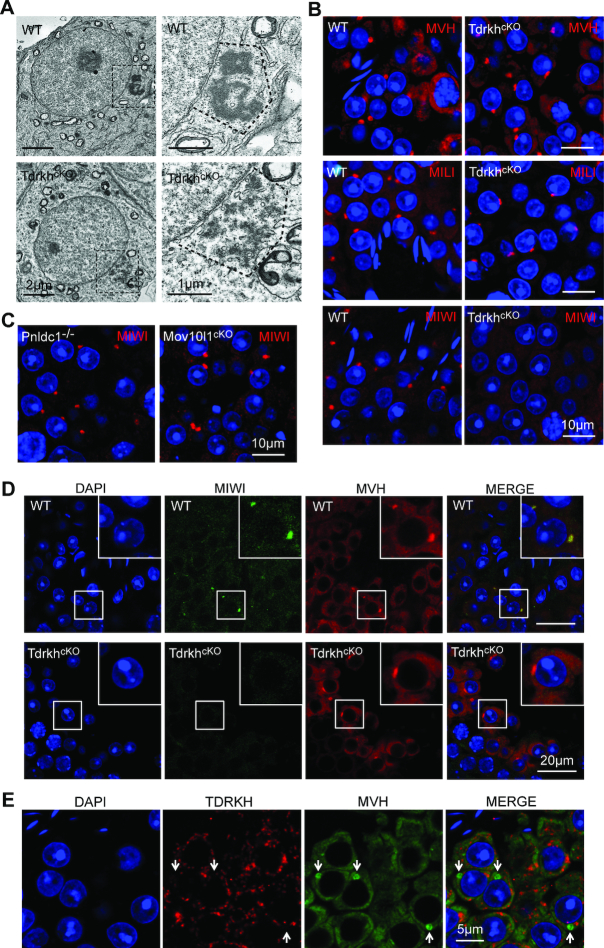
TDRKH deficiency disrupts the localization of MIWI but not MILI to chromatoid bodies in round spermatids. (**A**) TDRKH deficiency causes chromatoid body fragmentation in round spermatids. Transmission electron microscopy was performed on round spermatids from adult WT and *Tdrkh^cKO^* testes. The chromatoid bodies are indicated by dotted line and zoomed on the right. Scale bars, 2 μm (left) and 1 μm (right). (**B**) TDRKH deficiency specifically disrupts the localization of MIWI to the chromatoid body. Immunostaining was performed using MILI, MVH or MIWI antibody in round spermatids from adult WT and *Tdrkh^cKO^* testes. DNA was stained with DAPI. Scale bar, 10 μm. (**C**) PNLDC1 and MOV10L1 are not required for the localization of MIWI to the chromatoid body. Immunostaining was performed using MIWI antibody on adult *Mov10l1^cKO^* and *Pnldc1^−/−^* testes. DNA was stained with DAPI. Scale bar, 10 μm. (**D**) Loss of TDRKH disrupts the localization of MIWI to the chromatoid body. Immunostaining was performed using MIWI and MVH antibodies in round spermatids from adult WT and *Tdrkh^cKO^* testes. DNA was stained with DAPI. Scale bar, 20 μm. (**E**) TDRKH does not localize in chromatoid bodies in round spermatids. Immunostaining was performed using MVH and TDRKH antibodies in round spermatids from adult WT testes. DNA was stained with DAPI. Chromatoid bodes are indicated by white arrows. Scale bar, 5 μm.

Chromatoid body (CB) fragmentation in round spermatids is a common defect associated with piRNA deficiency observed in piRNA-deficient mutant mice (9,20,42). Similarly, we observed fragmented CB in *Tdrkh^cKO^* round spermatids (Figure 6A). Despite CB fragmentation, piRNA pathway proteins MVH and MILI were normally enriched at the CB in *Tdrkh^cKO^* round spermatids (Figure 6B). However, in stark contrast, MIWI were completely devoid from concentration in the CB, only exhibiting faint uniform cytoplasmic distribution (Figure 6B). The failure of MIWI localization to the CB is unique to TDRKH deficiency because MIWI was normally concentrated in the CB of *Pnldc1^−/−^* and *Mov10l1^cKO^* spermatids (Figure 6C) which displayed overall similar reduced MIWI expression level (Supplementary Figure S3), piRNA level (Supplementary Figure S4) and CB fragmentation (Supplementary Figure S9) (42). By co-immunostaining of MIWI and MVH, we confirmed the lack of MIWI in the CB *in situ* while the normal presence of MVH in the CB of the same *Tdrkh^cKO^* round spermatid (Figure 6D). Hence, TDRKH deficiency leads to a specific absence of MIWI but not MILI in the CB. This provides an unexpected genetic separation of differential compartmentalization of MIWI and MILI, suggesting distinct CB localization mechanism for these two PIWI members.

In wild-type round spermatids, TDRKH displays mitochondrial localization and is clearly not localized in the CB, because co-staining of TDRKH and MVH (CB concentrated) showed complete non-overlapping expression pattern (Figure 6E). This indicates that TDRKH is physically separated from the CB components including its binding partner MIWI despite their co-expression in round spermatids. This in turn indicates that TDRKH does not regulate MIWI localization in round spermatids. Together, our findings indicate that loss of MIWI in the CB of *Tdrkh^cKO^* round spermatids results from the failure of recruitment of MIWI to the IMC in *Tdrkh^cKO^* spermatocytes.

### Absence of MIWI in the CB is accompanied by defective transposon silencing in round spermatids

**Figure 7.**
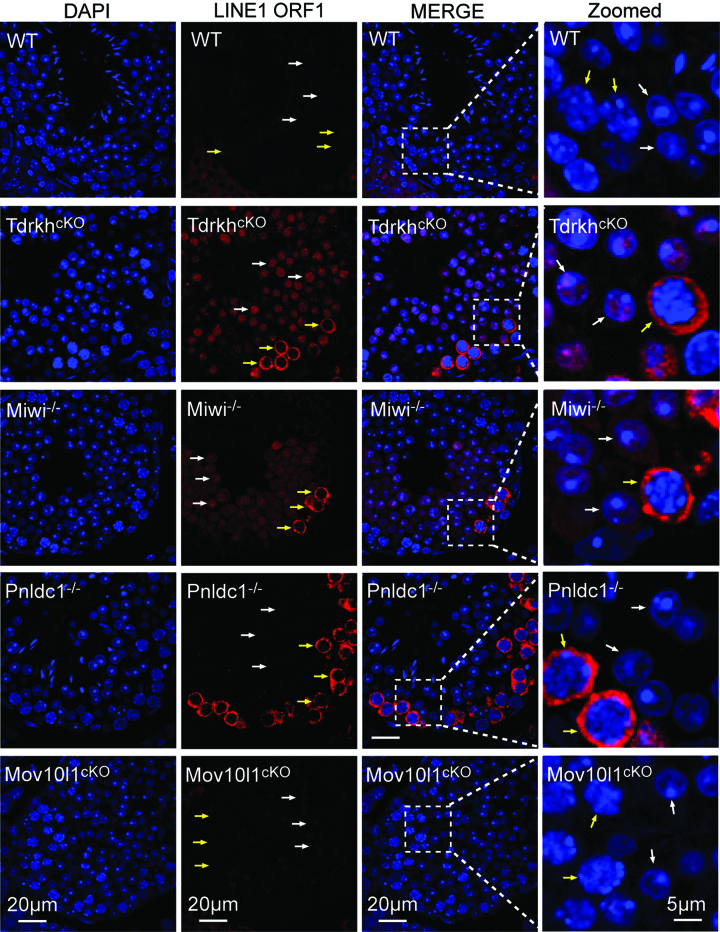
Absence of MIWI in the chromatoid body is accompanied by LINE1 de-repression in round spermatids. Immunostaining was performed using LINE1 ORF1 antibody on adult WT, *Tdrkh^cKO^, Miwi^−/−^, Pnldc1^−/−^* and *Mov10l1^cKO^* testes. DNA was stained with DAPI. Round spermatids are indicated by white arrows. Pachytene spermatocytes are indicated by yellow arrows. Magnified views are shown on the right panel.

We next examined the functional consequences of MIWI depletion (along with associated piRNAs) from CB by examining retrotransposon expression in *Tdrkh^cKO^* spermatids. It has been established that MIWI is the only PIWI protein required for LINE1 silencing in round spermatids while both MIWI and MILI are required for LINE1 suppression in spermatocytes (8,9). As a positive control, we observed LINE1 derepression in *Miwi^−/−^* spermatocytes and round spermatids (Figure 7). Similar to the pattern in *Miwi^−/−^*, LINE1 ORF1 was elevated in the cytoplasm of *Tdrkh^cKO^* spermatocytes and nuclei of *Tdrkh^cKO^* round spermatids (Figure 7). The derepression of LINE1 ORF1 in *Tdrkh^cKO^* round spermatids likely results from the depletion of MIWI in the CB rather than the loss of TDRKH in round spermatids. This is because TDRKH is unable to suppress LINE1 alone as evident by LINE1 upregulation in *Miwi^−/−^* spermatids that exhibited normal TDRKH expression and localization (Supplementary Figure S10A).

In *Mov10l1^cKO^* testes in which piRNAs are mostly depleted but normal CB localization of MIWI is maintained, LINE1 was properly silenced in both spermatocytes and round spermatids indicating that the bulk of piRNAs are not required for LINE1 repression in adult testes (42) (Figure 7). This in turn suggests that MIWI protein itself but not MIWI-piRNAs are critical for LINE1 silencing in round spermatids. In *Pnldc1^−/−^* testes, LINE1 was upregulated in spermatocytes but not in round spermatids, indicating that piRNA length maturation is not required for LINE1 repression in round spermatids (22) (Figure 7). Together, these data suggest the importance of MIWI CB localization in regulating transposon silencing in spermatids, and this function is likely piRNA-independent.

### TDRKH tethers the piRNA trimmer PNLDC1 to mitochondria

**Figure 8.**
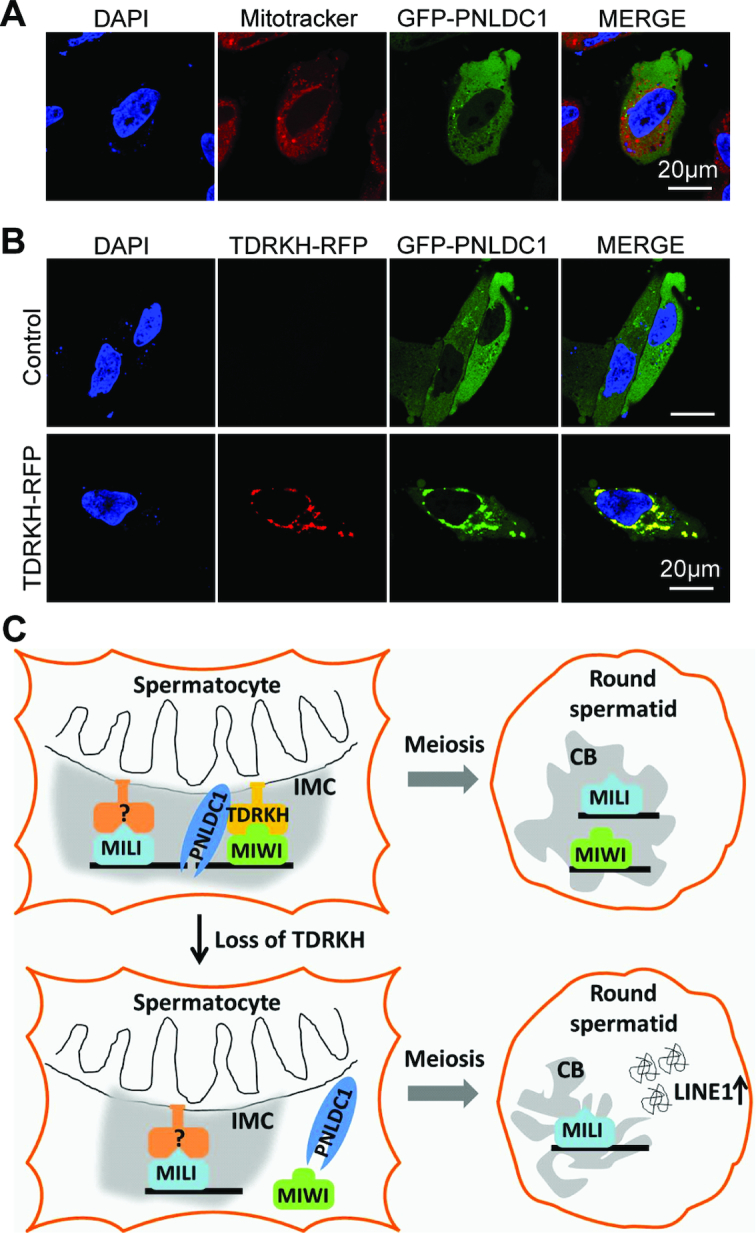
TDRKH tethers the trimmer PNLDC1 to mitochondria and a proposed model for TDRKH function. (**A**) PNLDC1 co-fluorescence with Mitotracker. HeLa cells were transfected with GFP-tagged PNLDC1 plasmids. After 48 h, the transfected cells were stained with Mitotracker and fixed. DNA was stained with DAPI. Scale bar, 20 μm. (**B**) TDRKH recruits PNLDC1 to mitochondria. HeLa cells were transfected with GFP-tagged PNLDC1 plasmids, along with indicated RFP-tagged plasmids. After 48 hours, the cells were fixed and DNA was stained with DAPI. Scale bar, 20 μm. (**C**) A proposed model illustrating the role of TDRKH in pachytene piRNA biogenesis and germ cell development. TDRKH specifically recruits MIWI to the IMC and an unknown protein anchors MILI at the IMC. Upon the piRNA intermediates loading into MIWI and MILI, TDRKH tethers the trimmer PNLDC1 to trim piRNA intermediates to final lengths. MIWI- and MILI-piRNA complexes mainly exist in the chromatoid body in round spermatids. MIWI represses LINE1 expression in chromatoid body likely through with its slicer activity in a piRNA-independent manner. MILI and its associated piRNAs are not sufficient to silence LINE1 in round spermatids.

TDRKH and PNLDC1 are tightly coupled in the piRNA trimming complex and the trimmer activity of PNLDC1 requires its partner with TDRKH (31). Since PNLDC1 has a putative C-terminal transmembrane motif, we hypothesized that PNLDC1, like TDRKH, self-localizes to mitochondrial membrane. To test this hypothesis, we expressed GFP- or Flag-tagged PNLDC1 in HeLa cells and analyzed its potential mitochondrial localization by co-staining with mitotracker. Interestingly, PNLDC1 was mainly diffused in cytoplasm and only slightly localize to mitochondria (Figure 8A and Supplementary Figure S11). This indicates that PNLDC1 has limited localizing ability to mitochondria in the HeLa cell system tested. Strikingly, when co-expressed with TDRKH, mitochondrial anchored TDRKH was able to drastically recruit and enrich PNLDC1 on mitochondria (Figure 8B). Consistent with this, PNLDC1 is not required for TDRKH mitochondrial localization, since TDRKH protein levels and mitochondrial localization was not disrupted in *Pnldc1^−/−^* spermatocytes (Supplementary Figure S10B). These results indicate that mitochondria-anchored TDRKH can recruit both PNLDC1 and MIWI to mitochondria and strongly suggest that TDRKH in spermatocytes plays a key role in the formation and function of the piRNA trimming complex in the IMC.

## DISCUSSION

In this study, we demonstrate that TDRKH acts as a key mitochondria-anchored scaffold protein that specifically recruits MIWI to the IMC and tethers PNLDC1 to couple two key piRNA biogenesis steps during pachytene piRNA biogenesis: MIWI recruitment and piRNA trimming. This TDRKH-mediated scaffolding function is essential for the production of MIWI-piRNAs, MIWI CB localization, transposon silencing and spermiogenesis. The discovery of TDRKH-dependent spatial segregation of MIWI and MILI in the IMC provides the molecular basis for understanding the functional differences between MIWI and MILI in transposon silencing and meiotic and postmeiotic germ cell development.

### Distinct mitochondrial localization mechanisms for MIWI and MILI

One key finding in this study is the spatial segregation of MIWI and MILI upon the loss of mitochondrial localized TDRKH in male germ cells. In TDRKH-deficient spermatocytes, MIWI is diffused while MILI remains enriched at the IMC. TDRKH strongly associates with MIWI, but less so with MILI (30,36). Our *in vivo* and *in vitro* studies demonstrate that TDRKH is both necessary and sufficient to engage MIWI in the piRNA biogenesis pathway. However, TDRKH is not required for the recruitment of MILI to the IMC. The simplest explanation for this is that MILI entry into the IMC is mediated by another protein, most likely a different mitochondria-anchored protein (Figure 8C). The identity of the putative MILI-specific binding protein needs further investigation.

The initial recruitment of MIWI by TDRKH is most likely in a native form without bound piRNAs. This is supported by the ability of TDRKH to enrich piRNA-free MIWI on mitochondria in the HeLa cell co-expression experiment (Figure 5). Based on our data, the recruitment of MIWI by TDRKH could be the first step to enrich MIWI at the mitochondrial surface, preparing MIWI for piRNA precursor binding, loading and trimming. TDRKH-mediated differential recruitment of MIWI and MILI provides a unique starting point to further dissect the complex piRNA processing mechanism in the IMC and the functional differences of MIWI and MILI, which are both essential for piRNA biogenesis and spermatogenesis.

### The TDRKH scaffolding function links MIWI recruitment to piRNA trimming

TDRKH is a mitochondria-localized protein tightly coupled with PNLDC1 to regulate piRNA trimming at the IMC (30,31). Although both TDRKH and PNLDC1 contain putative transmembrane regions, we found that TDRKH has the ability to self-target to mitochondrial membrane when transfected in cultured cells. The transmembrane region is required for its mitochondrial localization. In contrast, PNLDC1 has only weak mitochondrial localization potential, but is highly enriched at mitochondria when co-expressed with TDRKH (Figure 8). This indicates that TDRKH is the key mitochondria-anchored protein that docks PNLDC1 and MIWI to assemble the piRNA trimming complex for piRNA 3′ maturation. This partially explains why the trimmer activity of PNLDC1 is consistently dependent on the presence of TDRKH *in vitro* and *in vivo* (22,30,31).

Despite the inability of TDRKH to recruit MILI to mitochondria, the trimming of MILI-bound pre-piRNAs still requires TDRKH. This indicates that the PNLDC1/TDRKH trimming complex is accessible by pre-piRNA-bound MILI. PNLDC1 may exert the trimming activity through directly binding to piRNA-loaded PIWI proteins after complexed with TDRKH at the IMC. Indeed, PNLDC1 interacts with all PIWI proteins (MIWI, MILI and MIWI2) in an *in vitro* system (Supplementary Figure S12). The direct effect of TDRKH on MIWI protein localization and MIWI-piRNA production could explain why *Tdrkh^cKO^* mice have a more severe germ cell arrest than *Pnldc1^−/−^* mice. Thus, the mitochondria-anchored TDRKH protein complex not only facilities piRNA trimming but also involves initial recruitment of piRNA-free MIWI to the IMC, linking two important piRNA processing steps.

### Insight into mammalian chromatoid body formation and transposon silencing in spermatids

Although MIWI and MILI are both present in the CB of round spermatids, only MIWI is required for LINE1 silencing (8,9). This silencing depends on the slicer activity of MIWI (9), but is believed to be piRNA-independent (42). This is because in *Mov10l1^cKO^* mice, with piRNA-depleted MIWI in the CB, LINE1 is still properly silenced (42). Our data show that TDRKH deficiency causes selectively loss of MIWI from the CB and this correlates with LINE1 derepression in round spermatids, suggesting that MIWI CB residence is required for LINE1 repression. This in turn supports a critical role of CB in transposon silencing. The CB precursor formation in spermatocytes has been linked to PIWI-piRNA complexes derived from IMC (21). We propose that MIWI CB targeting is regulated by initial MIWI-TDRKH interaction at the IMC in pachytene spermatocytes, which is a prerequisite for MIWI residency in the CB of round spermatids. The requirement of TDRKH for round spermatid LINE1 silencing could be mediated by its requirement for MIWI to localize in the CB. We speculate that MIWI exerts its slicer activity in the CB to silence LINE1 expression. This piRNA-independent mechanism underlies the requirement of distinct silencing mechanisms in different germ cell types to safeguard the germline genome against transposons during multiple stages of spermatogenesis.

### Distinct mitochondrial TDRKH scaffolding protein complexes in diverse species

In *Drosophila*, among three PIWI family members (Piwi, Aub and Ago3), TDRKH/Papi specifically interacts with nuclear Piwi, but not with cytoplasmic Aub and Ago3 (34). Unlike in mice and silkworm, the PNLDC1/trimmer homolog does not exist in *Drosophila* (Supplementary Figure S13). However, TDRKH/Papi is required for Piwi-piRNA trimming despite the absence of PNLDC1/Trimmer (32). The mechanism by which TDRKH controls Piwi-piRNA trimming in *Drosophila* remains unknown. In the silkworm, TDRKH/Papi interacts with both cytoplasmic PIWI proteins, Siwi and Ago3, and is required for their piRNA 3′ end trimming by partnering with PNLDC1/Trimmer (31,44). This differs from the TDRKH/Papi scaffolding preference in mice, where TDRKH preferential binds MIWI, but not MILI, and genetically separates these two similar PIWI proteins during piRNA biogenesis. In *Drosophila* and silkworm, TDRKH/Papi only recognizes arginine methylated PIWI proteins (34,35,44,45). In mice, however, TDRKH interacts with MIWI in an arginine methylation-independent manner (33). Together, these studies reveal that although TDRKH/Papi is a scaffold protein generally required for piRNA trimming in different species, the molecular mechanisms by which TDRKH/Papi interacts with PIWI proteins and piRNA trimming enzymes to engage in the piRNA pathway vary across species.

## DATA AVAILABILITY

All sequencing data are deposited in the Sequence Read Archive of NCBI under the accession number SRP155835.

## Supplementary Material

Supplementary Data
