# Supplementary material for: Mitochondrial membrane-based initial separation of MIWI and MILI functions during pachytene piRNA biogenesis
Source: Nucleic Acids Res. 2018 Dec 22;47(5):2594–608. doi: 10.1093/nar/gky1281 (PMC6411938; doi:10.1093/nar/gky1281)
Supplement: Supplementary Data [file gky1281_supplemental_files.pdf]

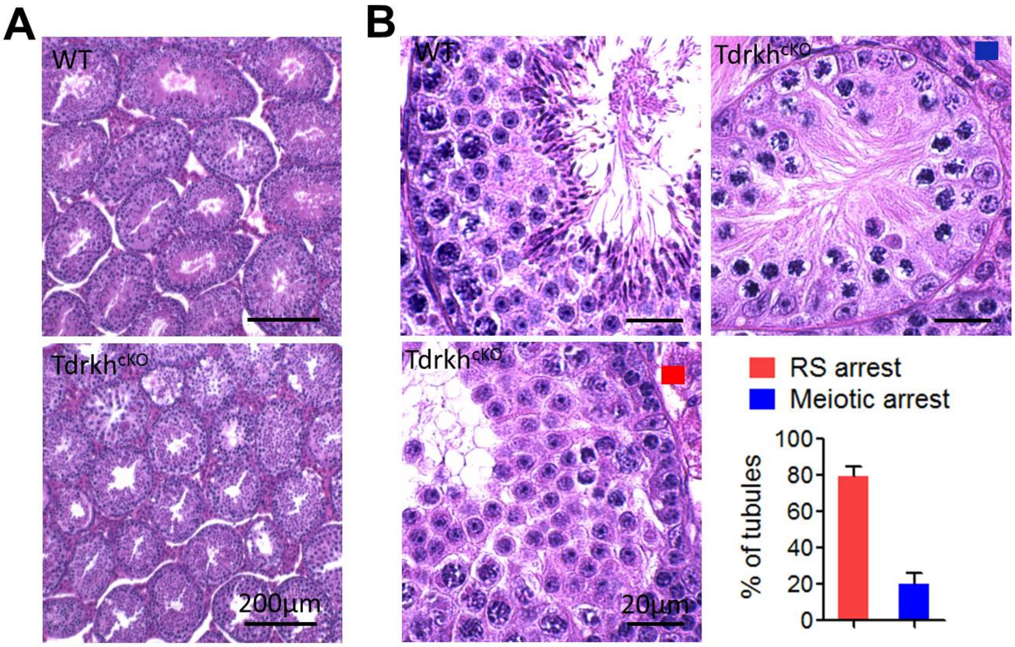

Figure S1. TDRKH deficiency in postnatal male germ cells causes spermatogenic arrest.
(A) Hematoxylin and eosin stained testis sections from adult WT and *Tdrkh*^{cKO} mice are shown. Scale bars, 200µm. (B) Different arrest stages observed in *Tdrkh*^{cKO} testes. Scale bars, 20µm. The percentage of round spermatid arrest and meiotic arrest seminiferous tubules in *Tdrkh*^{cKO} testes are shown. *Error bars* represent s.e.m. n=4.

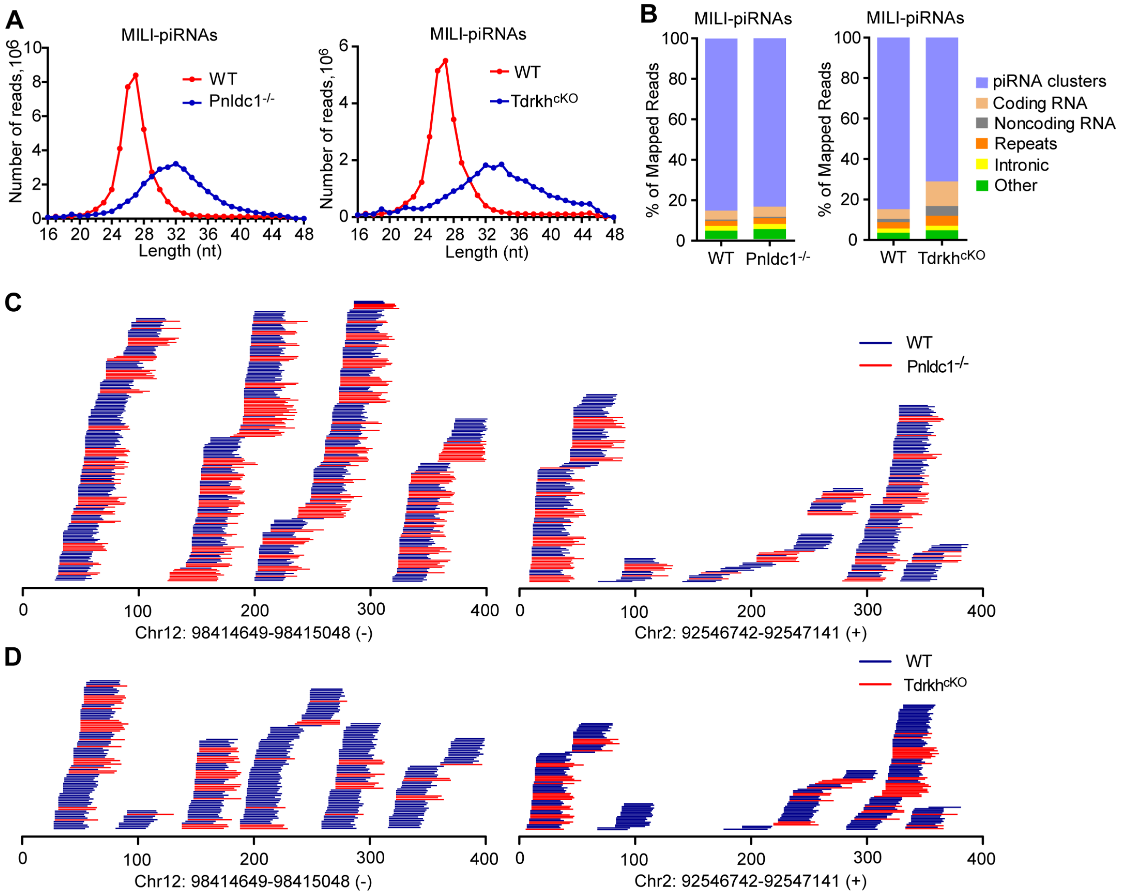

Figure S2. The 3' end extension of MILI-piRNAs in adult *Tdrkh*^{cKO} and *Pnldc1*$^{-/-}$ testes.

(A) The length distribution of MILI-piRNAs from adult WT, *Tdrkh*^{cKO} and *Pnldc1*$^{-/-}$ MILI-piRNA libraries.

(B) Genomic annotation of MILI-piRNAs from adult WT, *Tdrkh*^{cKO} and *Pnldc1*$^{-/-}$ testes. Sequence reads (24-48 nt) from MILI-piRNAs libraries were aligned to mouse genomic sequence sets in the following order: piRNA clusters, coding RNA, non-coding RNA, repeats, intronic sequences and other. The percentage of mapped reads is shown.

(C) Extended piRNA 3' ends in *Pnldc1*$^{-/-}$ testes. Alignments between 33-48 nt reads from *Pnldc1*$^{-/-}$ MILI-piRNA library and 24-32 nt reads from WT MILI-piRNA library within a selected regions from two representative piRNA clusters. The genomic locations of the piRNA clusters are shown at the bottom.

(D) Extended piRNA 3' ends in *Tdrkh*^{cKO} testes. Alignments between 31-48 nt reads from *Tdrkh*^{cKO} MILI-piRNA library and 24-30 nt reads from WT MILI-piRNA library within a selected regions from two representative piRNA clusters. The genomic locations of the piRNA clusters are shown at the bottom.

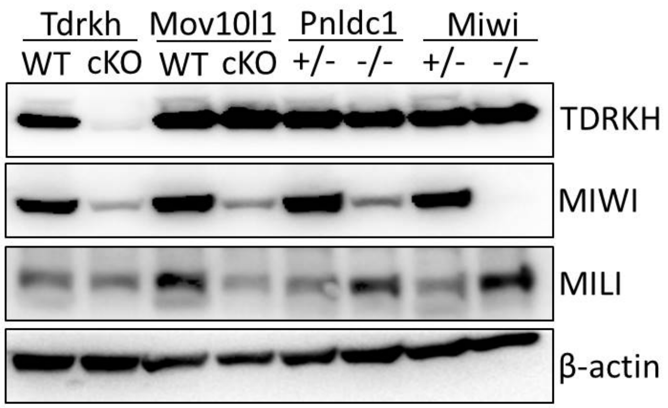

Figure S3. Protein expression of TDRKH, MILI and MIWI in *Tdrkh*^{cKO}, *Mov10l1*^{cKO}, *Pnldc1*$^{-/-}$ and *Miwi*$^{-/-}$ mice. Western blotting showing the expression of TDRKH, MILI and MIWI in testes from indicated mice. β-actin served as a loading control.

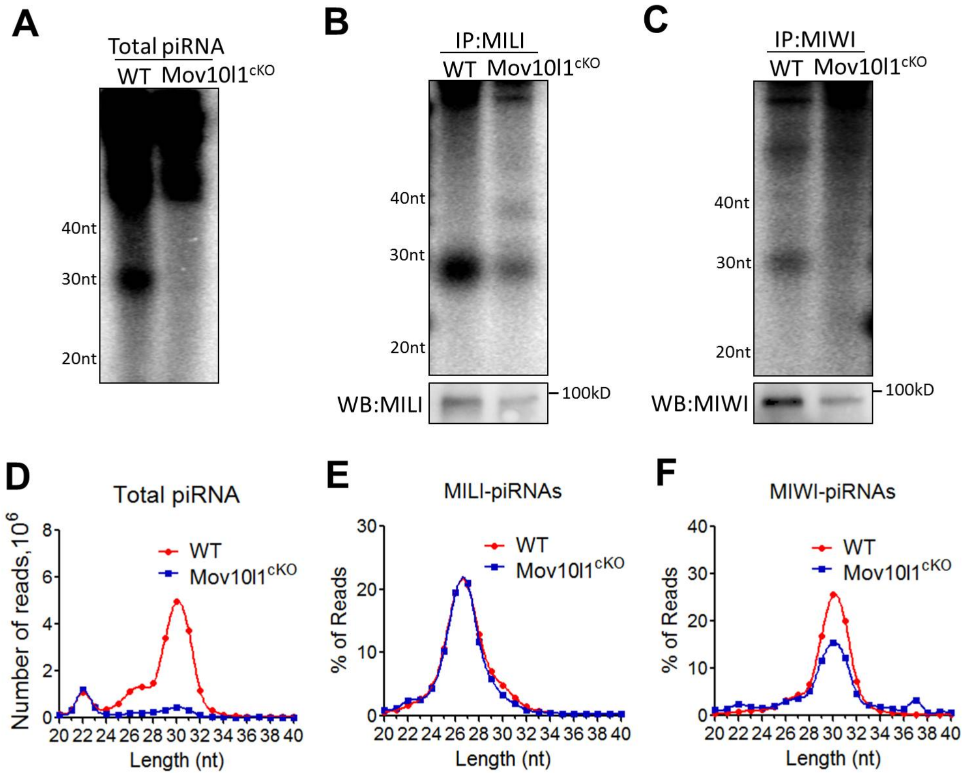

Figure S4. MOV10L1 deficiency in postnatal germ cells causes pachytene piRNA defects in adult testes.

(A) Reduction of total piRNA in *Mov10l1^{cKO}* testes. Total RNAs from adult WT and *Mov10l1^{cKO}* testes were end-labeled with [^{32}P]-ATP and detected by 15% TBE urea gel and autoradiography.

(B) Reduction of MILI-piRNA in *Mov10l1^{cKO}* testes. Small RNAs were isolated from immunoprecipitated MILI RNPs and were end-labeled with [^{32}P]-ATP and detected by 15% TBE urea gel and autoradiography. Western blotting was performed with anti-MILI antibody to show immunoprecipitation efficiency.

(C) Diminished MIWI-piRNA in *Mov10l1^{cKO}* testes. Small RNAs were isolated from immunoprecipitated MIWI RNPs and were end-labeled with [^{32}P]-ATP and detected by 15% TBE urea gel and autoradiography. Western blotting was performed with anti-MIWI antibody to show immunoprecipitation efficiency.

(D) The length distribution of small RNAs from adult WT and *Mov10l1^{cKO}* testicular small RNA libraries. Data were normalized by miRNA reads (21nt-23nt).

(E) The length distribution of MILI-piRNAs from adult WT and *Mov10l1^{cKO}* MILI-piRNA libraries.

(F) The length distribution of MIWI-piRNAs from adult WT and *Mov10l1^{cKO}* MIWI-piRNA libraries.

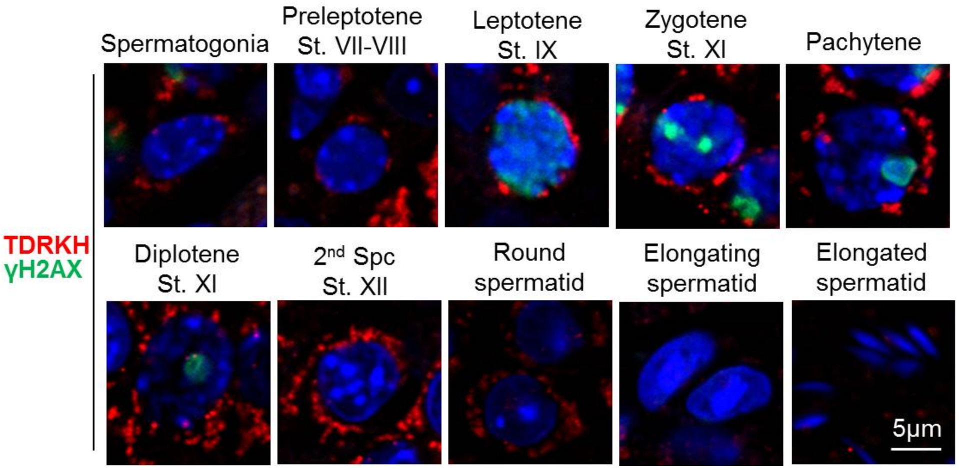

Figure S5. TDRKH protein localization in the mouse testis.
Co-immunostaining was performed using TDRKH and γH2AX antibodies on adult WT testis sections. Different germ cell types were distinguished according to γH2AX staining and DAPI staining. Scale bar, 5μm.

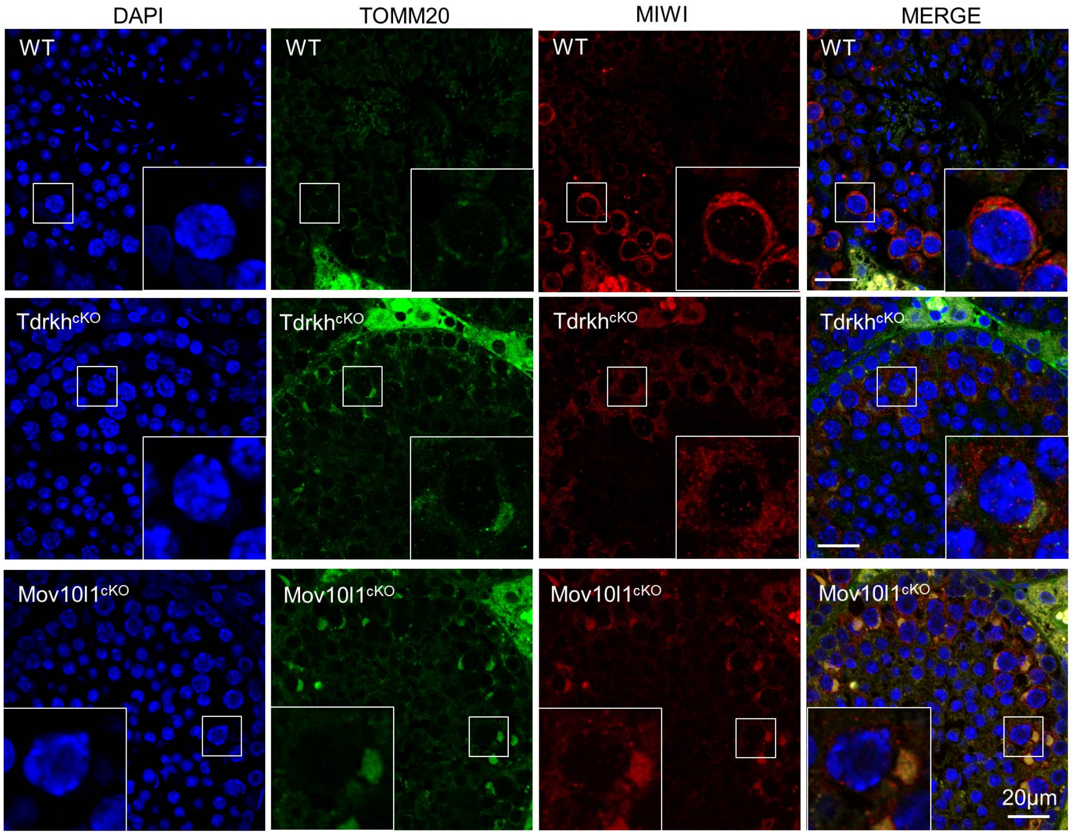

Figure S6. TDRKH deficiency disrupts the localization of MIWI to mitochondria.
Co-immunostaining was performed using MIWI and TOMM20 antibodies on adult WT, *Tdrkh*^{cKO} and *Mov10l1*^{cKO} testes. DNA was stained with DAPI. TOMM20 served as a mitochondrial marker. Scale bar, 20µm.

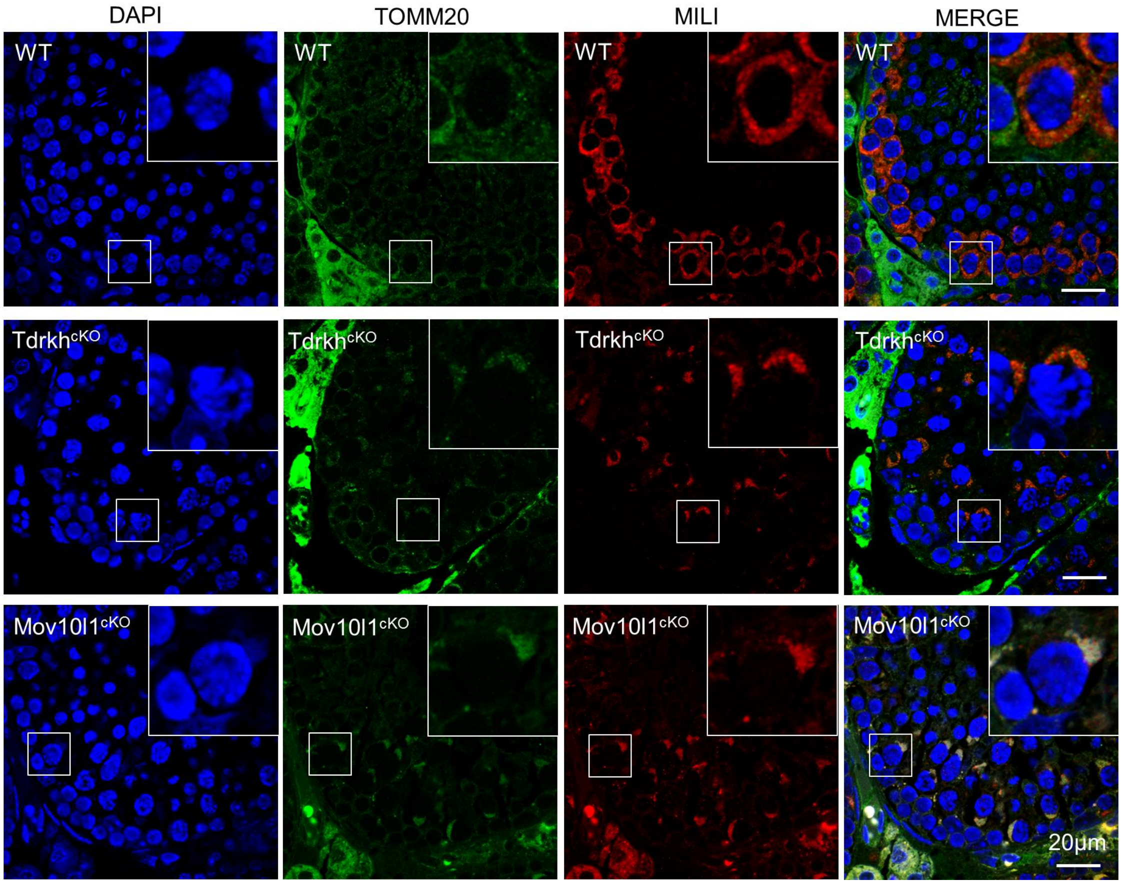

Figure S7. The mitochondrial aggregation of MILI in *Tdrkh*^{cKO} and *Mov10l1*^{cKO} testes.
Immunostaining was performed using MILI and TOMM20 antibodies on adult WT, *Tdrkh*^{cKO} and *Mov10l1*^{cKO} testes. DNA was stained with DAPI. TOMM20 served as a mitochondrial marker. Scale bar, 20μm.

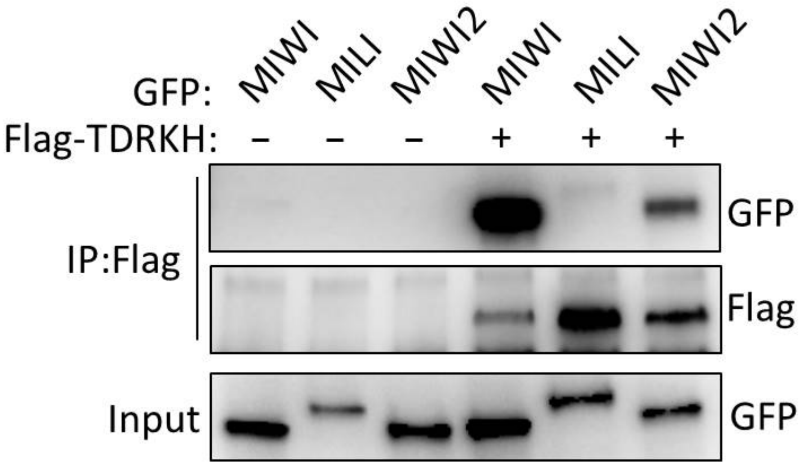

Figure S8. TDRKH interacts with MIWI and MIWI2, but minimally with MILI.
HEK293T cells were transfected with indicated plasmids. After 48 hours, immunoprecipitations were performed using anti-Flag resin. GFP-tagged proteins and Flag-TDRKH were detected with GFP and Flag antibodies by Western blotting.

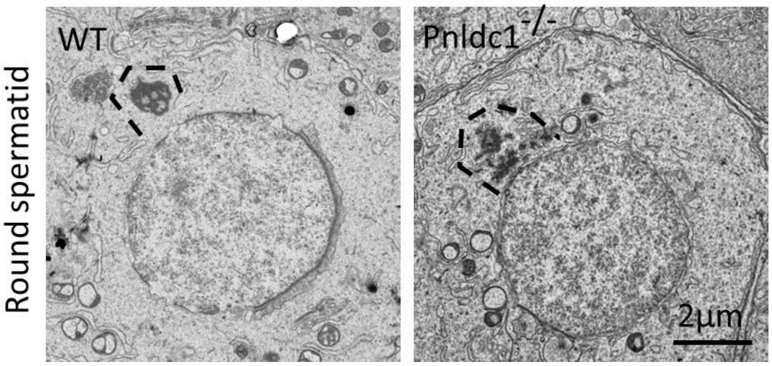

Figure S9. PNLDC1 deficiency causes chromatoid body fragmentation.
Transmission electron microscopy was performed on round spermatids from adult WT and *Pnldc1*$^{-/-}$ testes. The chromatoid bodies were indicated by dotted line. Scale bar, 2μm.

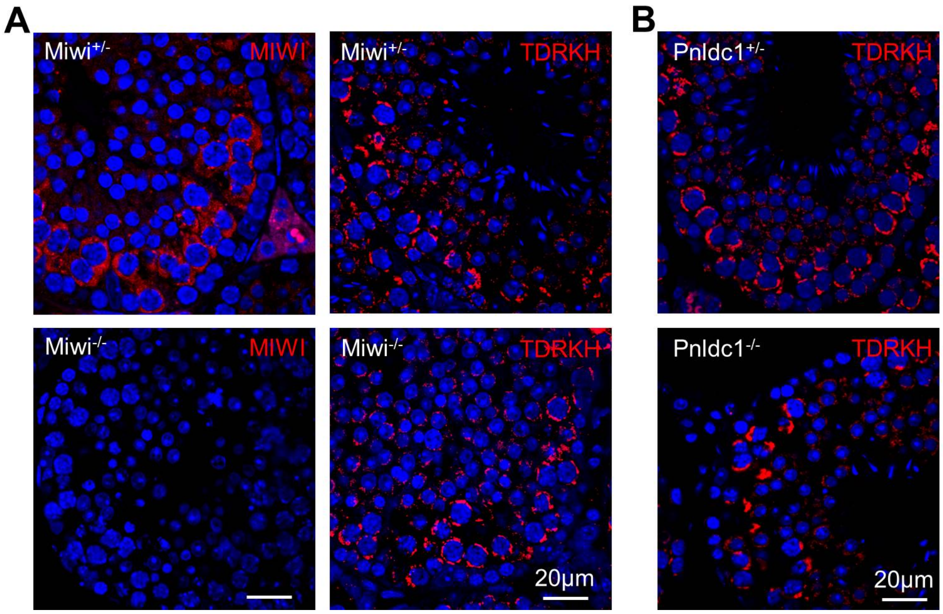

Figure S10. TDRKH protein localization in *Pnldc1$^{-/-}$* and *Miwi$^{-/-}$* testes.

(A) MIWI deficiency does not affect the localization of TDRKH. Immunostaining was performed using MIWI and TDRKH antibodies on adult *Miwi$^{+/-}$* and *Miwi$^{-/-}$* testes. DNA was stained with DAPI. Scale bar, 20μm.

(B) PNLDC1 deficiency does not affect TDRKH mitochondrial localization. Immunostaining was performed using TDRKH antibody on adult *Pnldc1$^{+/-}$* and *Pnldc1$^{-/-}$* testes. DNA was stained with DAPI. Scale bar, 20μm.

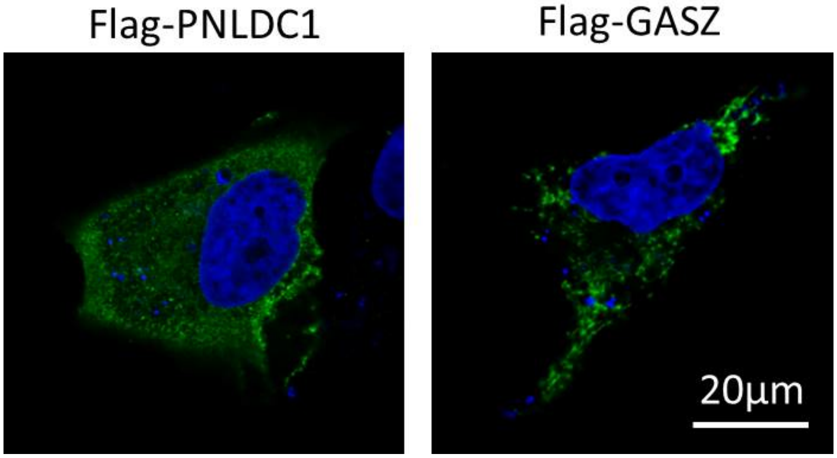

Figure S11. Intracellular localization of PNLDC1 in HeLa cells.
HeLa cells were transfected with indicated Flag-tagged plasmids. After 48 hours, the transfected cells were fixed and immunostained with Flag antibody. DNA was stained with DAPI. Scale bar, 20μm. GASZ served as a positive control for mitochondrial localization. PNLDC1 did not show typical mitochondrial localization pattern.

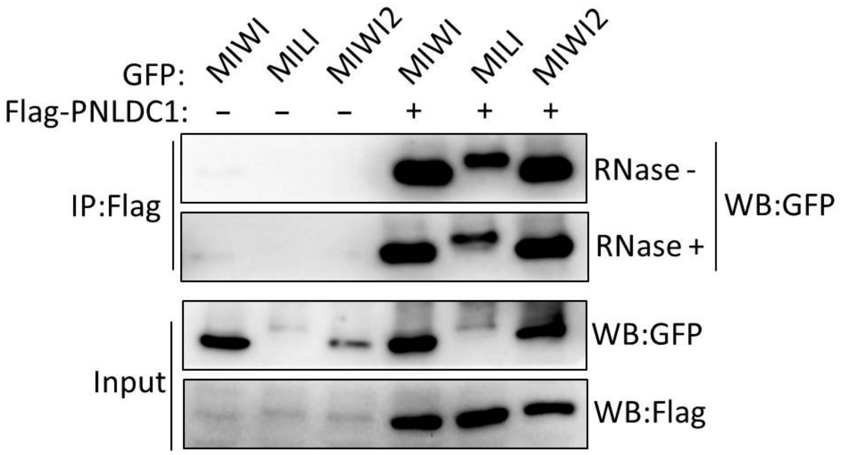

Figure S12. PNLDC1 interacts with MIWI, MILI and MIWI2.
HEK293T cells were transfected with indicated plasmids. After 48 hours, the cell lysates were treated with or without RNase A. Immunoprecipitations were performed using anti-Flag resin. GFP-tagged PIWI proteins and Flag-PNLDC1 were detected with GFP and Flag antibodies by Western blotting.

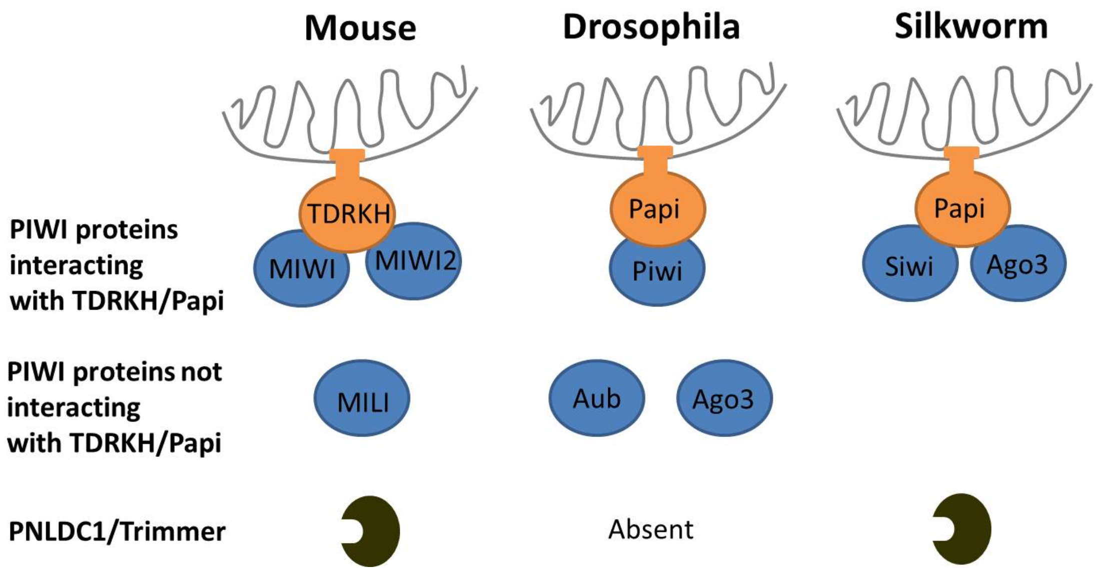

Figure S13. Interaction relationship between TDRKH/Papi and PIWI proteins among different species. PNLDC1 has conserved binding with mouse and silkworm TDRKH/papi. However, the PNLDC1 gene is absent in the *Drosophila* genome.
